# A Low Noise Amplifier for Neural Spike Recording Interfaces

**DOI:** 10.3390/s151025313

**Published:** 2015-09-30

**Authors:** Jesus Ruiz-Amaya, Alberto Rodriguez-Perez, Manuel Delgado-Restituto

**Affiliations:** Institute of Microelectronics of Seville, Avda. Americo Vespucio s/n, Sevilla 41092, Spain; E-Mails: ruiz@imse-cnm.csic.es (J.R.-A.); alberto@imse-cnm.csic.es (A.R.-P.)

**Keywords:** Low-Noise Amplifier, neural spike recording, biomedical circuit, circuit sizing

## Abstract

This paper presents a Low Noise Amplifier (LNA) for neural spike recording applications. The proposed topology, based on a capacitive feedback network using a two-stage OTA, efficiently solves the triple trade-off between power, area and noise. Additionally, this work introduces a novel transistor-level synthesis methodology for LNAs tailored for the minimization of their noise efficiency factor under area and noise constraints. The proposed LNA has been implemented in a 130 nm CMOS technology and occupies 0.053 mm-sq. Experimental results show that the LNA offers a noise efficiency factor of 2.16 and an input referred noise of 3.8 *μ*Vrms for 1.2 V power supply. It provides a gain of 46 dB over a nominal bandwidth of 192 Hz–7.4 kHz and consumes 1.92 *μ*W. The performance of the proposed LNA has been validated through *in vivo* experiments with animal models.

## 1. Introduction

During the last years, there has been a growing interest on the design of implanted neural recording interfaces for the monitoring of brain activity [1,2,3,4,5,6,7,8,9,10,11,12,13,14,15,16,17,18,19,20,21]. The information acquired by these interfaces can be used for the prevention and treatment of many neural diseases, as well as in Brain Machine Interfaces (BMIs) [22,23,24]. Typically, a large population of neurons has to be simultaneously monitored in these applications (in some recent implementations around 500 recording sensors are used [25]), thus leading to highly complex circuit solutions. In spite of this complexity, neural prosthesis has to exhibit low power consumption, in order to avoid excessive heating of the brain tissue [26], and preserve a small form factor.

**Figure 1:**
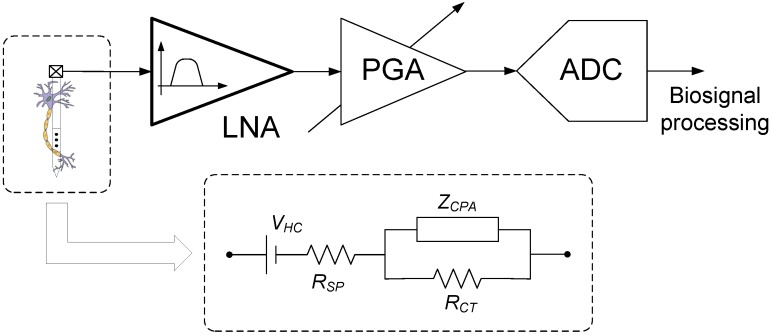
Simplified diagram of a typical neural recording channel and electrical model of the tissue-microelectrode interface (inset).

As shown in Figure 1, a typical recording sensor is composed by a microelectrode to capture the neural activity, followed by a Low Noise Amplifiers (LNA), a Programmable Gain Amplifier (PGA), and an Analogue-to-Digital Converter (ADC) to digitize the acquired data for further profcessing. The PGA is tailored for amplifying the signal coming from the LNA, which commonly offers a fixed voltage gain in the range from 30 to 50 dB, so as to maximally cover the input range of the following ADC. In this scheme, the LNA is often responsible for the main area and power consumptions. When tailored to the acquisition of neural action potentials by means of intracortical microelectrodes, the LNA must be able to boost the weak spike events of few tens of $\mu V^{\prime }s$ detected by the probe and filter out the undesired frequency components. This demands the use of circuit topologies with low input-referred noise while keeping area and power consumptions small.

Being the first element in the readout circuitry of the neural recording sensor, the LNA must also satisfy other requirements arising from the particular characteristics of the tissue-microlectrode interface. As shown at the inset of Figure 1, such interface is commonly modeled by a double-layer capacitance with constant phase angle impedance $Z_{CPA}$ (which measures the non-faradaic charge transfer at the boundary between the electrode and the tissue), shunted by a charge transfer resistance $R_{CT}$ (which represents the faradaic process where charges transfer between the electrode and the tissue by means of oxidation–reduction reactions), in series with a spreading resistance $R_{SP}$ (which models the resistance of the tissue and depends on the geometrical area of the electrode) [27,28]. In data sheets of commercial intracortical microelectrodes, this reactive behavior is often summarized by the mean 1 kHz impedance, $Z_{1kHz}$ (this is the fundamental action potential frequency often used to probe tissue properties around an implanted microelectrode). Results from different microelectrode arrays available in the market show that such $Z_{1kHz}$ usually falls below 200kΩ [29]. In order to preclude a substantial signal attenuation due to voltage division effects, the input impedance of the LNA has to be much larger than the tissue-microlectrode impedance.

Another concern is the steady potential, called half-cell potential, generated between the electrode and the tissue as a consequence of gradients in the ion-electron exchange through the interface [30]. This half-cell potential, represented in Figure 1 by a DC source $V_{HC}$, is typically several hundred mV’s and it is dependent on the material of the microelectrode and the size and shape of the recording site. The half-cell potential can only be measured with respect to another electrode which acts as reference. The mismatch in half-cell potentials between the reference and the recording electrodes is responsible for a differential DC offset voltage at the input of the LNA. The magnitude of this DC offset can be as large as 1–2 V and, hence, it may swamp the much smaller neural signals to be measured [2]. Obviously, to prevent the LNA from saturation, circuit techniques have to be provided for offset blocking. It is worth mentioning this offset voltage does not provide a completely stable baseline but actually drifts, thus introducing low frequency components into the monitored biosignal [31]. This is particularly problematic for the recording of local field potentials which extends down to few Hz’s. In order to overcome this problem, sophisticated circuit techniques such as chopping, auto-zeroing or DC servo loops have to be incorporated in the design of the LNA [32,33,34,35]. In neural spike recording sensors, DC drifting effects can be filtered out more easily given than the bandwidth of interest typically lies between 200 Hz and 7 kHz [36].

In this paper, five of the most common LNA topologies suitable for neural spike recording are reviewed [2,3,6,7,8,13,15,18] and, afterward, a novel solution based on a two-stage structure with feed-forward compensation technique is presented. It is analytically demonstrated that the presented structure obtains a 40 dB/dec magnitude roll-off in the low-pass transfer characteristic, which allows to reduce the in-band integrated noise as compared to prior art. The proposed topology has been sized by means of an optimization routine aiming to reduce its Noise Efficiency Factor (NEF) under area and power consumption constraints. Typical specifications for the recording of neural spikes are targeted. To illustrate the versatility of the sizing approach, the reviewed LNA topologies has also been dimensioned for the same circuit requirements. It is shown that the reported proposal improves by about 15% the NEF value over one of the best topologies reported so far [18], with negligible impact in area and power consumptions.

The proposed LNA has been fabricated in a 130 nm standard CMOS technology. It provides a midband gain of 46 dB over the recording bandwidth using a supply voltage of 1.2 V. The circuit consumes 1.92 *μ*W and obtains an input referred noise of $3.8\;\mu V_{rms}$, resulting in a NEF of only 2.16. The proposed LNA uses a fully-differential structure able to provide high common mode and power supply rejection ratios (above 75 dB in both cases) as well as a good linearity performance (higher than 60 dB total harmonic distortion for $3\;mV_{pp}$ input signal levels). *In vivo* results with a rat model using penetrating microelectrodes validate the performance of the LNA and confirm its suitability for neural spike recording.

## 2. LNA Topology Study

**Figure 2:**
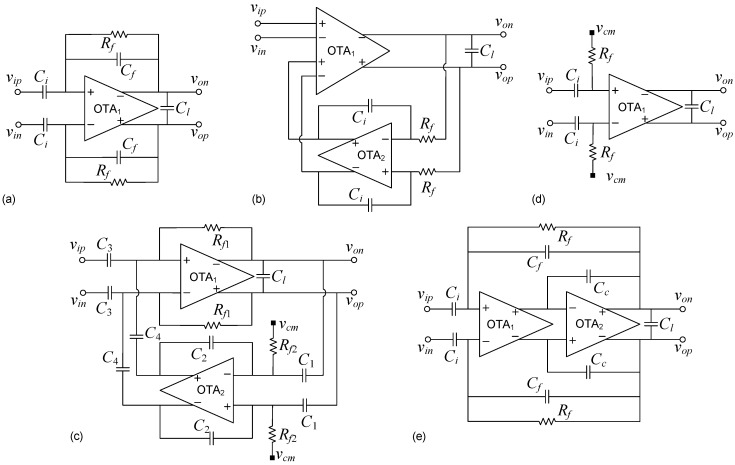
LNA architectures reviewed: (**a**) CFN; (**b**) MIFN; (**c**) CAFN; (**d**) OLN; and (**e**) MCCFN approaches.

Figure 2 shows five popular LNA topologies typically used for neural acquisition interfaces. They are referred to as Capacitive Feedback Network (CFN) [2,17,37], Miller Integrator Feedback Network (MIFN) [3], Capacitive Amplifier Feedback Network (CAFN) [6], Open Loop Network (OLN) [38] and Miller Compensated Capacitive Feedback Network (MCCFN) [8,18,39]. Fully-differential structures have been considered for their robustness against supply and common-mode voltage variations although the following discussion can be straightforwardly applied to single-ended topologies.

With the exception of the MIFN topology, the LNAs in Figure 2 use a DC blocking input capacitor $C_{i}$ for offset cancellation. This AC coupling capacitor, typically in the order of 20 to 30 pF, dominates the input impedance $Z_{in}$ of the structure and makes it more than one order of magnitude larger than the overall impedance of the tissue-microlectrode interface. This induces a small attenuation on the acquired signal which can be easily compensated by the following PGA (see Figure 1). The MIFN structure does not use AC coupling but employs a low-frequency suppression technique in which the input parasitic capacitance of the direct-path Operational Transconductance Amplifier (OTA) dominates $Z_{in}$ [3,40]. As this parasitic capacitance is typically in the order of few pF’s, lower levels of signals attenuation can be expected with the MIFN topology.

Table 1 summarizes the transfer characteristics and noise performances of these topologies obtained after small-signal analysis. In this table, single-pole networks have been considered for the OTAs which are thus characterized by a transconductance $g_{m}$, output conductance $g_{o}$ (the DC-gain of the OTA is given by $A_{o}=g_{m}/g_{o}$) and input and output capacitances, $C_{pi}$ and $C_{po}$, respectively. Subindexes 1 and 2 are used to distinguish between $OTA_{1}$ and $OTA_{2}$ where apply. Assuming in all cases that $g_{m1,2}R_{f}\gg 1$, $A_{o1,2}\gg 1$ and that the non-dominant poles and zeros are at high frequencies (conditions are expressed in the third column of Table 1 along with definitions of some intermediate variables), the five topologies feature a bandpass transfer characteristic which, in the frequency range of interest, can be approximated as:

(1) $$H\left(s\right)≃\frac{G(1+\frac{s}{z_{1}})}{\left(1+\frac{s}{p_{1}}\right)\left(1+\frac{s}{p_{2}}\right)}$$

**Table 1:** LNA topologies performance comparison.

Topology	Transfer Function Parameters	Variables and Conditions	Noise Performance
CFN [2]	$\begin{array}{c} G\approx -C_{i}R_{f}\;\;\;\;M_{bg}\approx -\frac{C_{i}}{C_{f}}\\ z_{1}\approx 0\\ p_{1}\approx \frac{-1}{R_{f}C_{f}}\;\;\;\;\;p_{2}\approx \frac{-g_{m}}{A_{o}C_{eq}} \end{array}$	$\begin{array}{c} \beta =\frac{C_{f}}{C_{pi}+C_{i}+C_{f}}\\ C_{t}=C_{l}+C_{po}\\ \begin{array}{c} C_{eq}=C_{pi}+C_{i}+C_{t}/\beta \end{array}\\ A_{o}\gg 1/\beta \end{array}$	$\begin{array}{c} \begin{array}{c} v_{rms}\approx \sqrt{\frac{KT\gamma }{M_{bg}}\left(\frac{1}{C_{i}}+\frac{n(1+\eta )}{2C_{t}}\right)} \end{array} \end{array}$
			$\begin{array}{c} \begin{array}{c} NEF\geq n\sqrt{\frac{\gamma k\left(1+\eta \right)}{2}} \end{array} \end{array}$
MIFN [3]	$\begin{array}{c} G\approx -1/A_{o2}\;\;\;\;M_{bg}\approx -A_{o1}\\ z_{1}\approx \frac{-1}{A_{o2}C_{i}R_{f}}\\ p_{1}\approx \frac{-A_{o1}}{R_{f}C_{i}}\;\;\;\;p_{2}\approx \frac{-g_{m1}}{A_{o1}C_{t1}} \end{array}$	$\begin{array}{c} \beta_{2}=\frac{C_{i}}{C_{pi2}+C_{i}}\\ C_{t1}=C_{po1}+C_{l}\\ C_{t2}=C_{pi1}+C_{po2}\\ C_{eq2}=C_{pi2}+\frac{C_{t2}}{\beta_{2}}\\ \alpha =\frac{g_{m2}}{g_{m1}}\gg \frac{C_{eq2}}{A_{o1}C_{t1}} \end{array}$	$\begin{array}{c} \begin{array}{c} v_{rms}\approx \sqrt{\frac{KT\gamma }{M_{bg}}\left(\frac{1}{C_{i}}+\frac{n(2+\eta_{1}+\psi_{2})}{2C_{t1}}\right)} \end{array}\\ \psi_{2}=\frac{1+\eta_{2}}{\beta_{2}^{2}\alpha } \end{array}$
			$\begin{array}{c} \begin{array}{c} NEF\geq n\sqrt{\frac{\gamma \left(k_{1}+k_{2}\alpha \right)\left(2+\eta_{1}+\frac{1+\eta_{2}}{\alpha }\right)}{2}} \end{array} \end{array}$
CAFN [6]	$\begin{array}{c} G\approx -C_{3}R_{f1}\;\;\;\;M_{bg}\approx \frac{-C_{2}C_{3}}{C_{1}C_{4}}\\ z_{1}\approx 0\\ p_{1}\approx \frac{-C_{2}}{R_{f1}C_{1}C_{4}}\;\;\;\;p_{2}\approx \frac{-g_{m1}\beta_{1}C_{1}}{C_{2}C_{t1}} \end{array}$	$\begin{array}{c} \beta_{1}=\frac{C_{4}}{C_{3}+C_{4}+C_{pi1}}\\ \beta_{2}=\frac{C_{2}}{C_{pi2}+C_{1}+C_{2}}\\ C_{t1}=C_{po1}+C_{l}+C_{1}\\ C_{eq2}=C_{pi2}+C_{1}+\frac{C_{po2}+C_{4}}{\beta_{2}}\\ A_{o1}\gg \frac{C_{3}+C_{p1}}{\beta_{2}C_{1}}\;\;\;\;A_{o2}\gg \frac{1}{\beta_{2}}\\ \alpha =\frac{g_{m2}}{g_{m1}}\gg \frac{\beta_{1}C_{1}}{C_{t1}} \end{array}$	$\begin{array}{c} v_{rms}\approx \sqrt{\frac{KT\gamma }{M_{bg}}\left(\frac{1}{C_{3}}+\frac{n(1+\eta_{1}+\chi_{2})}{2C_{t1}}\right)}\\ \chi_{2}=\frac{\left(1+\eta_{2}\right)\beta_{1}^{2}}{\beta_{2}^{2}\alpha } \end{array}$
			$\begin{array}{c} NEF\geq \begin{array}{c} n\sqrt{\frac{\gamma k_{1}\left(1+\eta_{1}\right)}{2}} \end{array} \end{array}$
OLN [38]	$\begin{array}{c} G\approx -A_{o}C_{i}R_{f}\;\;\;\;M_{bg}\approx -A_{o}\beta \\ z_{1}\approx 0\\ p_{1}\approx \frac{-\beta }{R_{f}C_{i}}\;\;\;\;p_{2}\approx \frac{-g_{m}}{A_{o}C_{t}} \end{array}$	$\begin{array}{c} \beta =\frac{C_{i}}{C_{pi}+C_{i}}\\ C_{t}=C_{po}+C_{l}\\ C_{eq}=C_{pi}+C_{i}+\frac{C_{t}}{\beta } \end{array}$	$\begin{array}{c} v_{rms}\approx \sqrt{\frac{KT\gamma }{M_{bg}}\left(\frac{A_{o}}{C_{i}}+\frac{n(1+\eta )}{2\beta C_{t}}\right)} \end{array}$
			$\begin{array}{c} \begin{array}{c} NEF\geq \frac{n}{\beta }\sqrt{\frac{\gamma k}{2}\left(1+\eta +\frac{2A_{o}C_{t}}{nC_{i}}\right)} \end{array} \end{array}$
MCCFN [18]	$\begin{array}{c} G\approx -C_{i}R_{f}\;\;\;\;M_{bg}\approx -C_{i}/C_{f}\\ z_{1}\approx 0\\ p_{1}\approx \frac{-1}{R_{f}C_{f}}\;\;\;\;p_{2}\approx \frac{-\beta g_{m1}}{C_{c}} \end{array}$	$\begin{array}{c} \beta =\frac{C_{f}}{C_{pi1}+C_{i}+C_{f}}\\ C_{t1}=C_{po1}+C_{pi2}\\ C_{t2}=C_{po2}+C_{l}\\ C_{eq}=C_{pi1}+C_{i}+C_{t2}/\beta \\ A_{o2}\gg \frac{C_{t1}}{C_{c}}\\ \alpha =\frac{g_{m2}}{g_{m1}}\gg \frac{\beta C_{eq}}{C_{c}A_{o1}} \end{array}$	$\begin{array}{c} \begin{array}{c} v_{rms}\approx \sqrt{\frac{KT\gamma }{M_{bg}}\left(\frac{1}{C_{i}}+\frac{n(1+\eta_{1})}{2C_{c}}\right)} \end{array} \end{array}$
			$\begin{array}{c} NEF\geq n\sqrt{\frac{\gamma k_{1}\left(1+\eta_{1}\right)}{2}} \end{array}$

**Figure 3:**
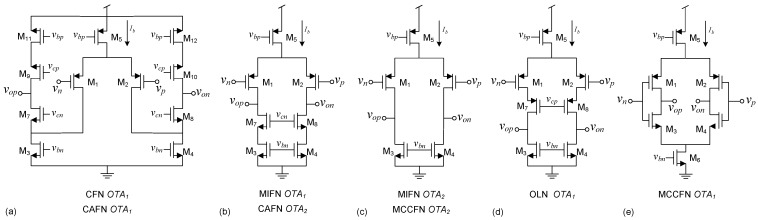
Transistor-level OTA implementation for: (**a**) $OTA{}_{1}$ in CFN and CAFN; (**b**) $OTA{}_{1}$ in MIFN and $OTA{}_{2}$ in CAFN; (**c**) $OTA_{2}$ in MIFN and MCCFN; (**d**) $OTA_{1}$ in OLN; (**e**) $OTA_{1}$ in MCCFN architectures.

In Euquation (1) $p_{1}$ and $p_{2}$ represent the high- and low-pass poles, respectively, and $z_{1}$ is a zero close to the origin ($z_{1}\ll p_{1}\ll p_{2}$). Their values, together with the passband midgain $M_{bg}$, are expressed in the second column of Table 1. The fourth column illustrates the thermal noise performance of the LNA topologies including the input-referred rms noise $v_{rms}$ and the noise efficiency factor NEF, defined as [41]:

(2) $$NEF=v_{rms}\cdot \sqrt{\frac{2I_{tot}}{\pi \cdot U_{t}\cdot 4KT\cdot BW}}$$

where $U_{t}=KT/q$ is the thermal voltage, *K* is the Boltzmann’s constant, *T* is the absolute temperature, *q* is the electron charge, $I_{tot}$ is the total current consumption of the LNA, and BW stands for its 3 dB-bandwidth. Note that this paper focuses exclusively on thermal noise contributions. Flicker noise may also impact in the noise characteristics of the LNAs, but it can be substantially reduced by using large transistor dimensions or chopper or auto-zero techniques. In Table 1 it is assumed that the total current consumption is proportional to the bias current $I_{b}$ of the input differential pair of the OTA, *i.e.*, $I_{tot}=k\cdot I_{b}$, where *k* depends on the particular OTA topology and accounts for the biasing circuitry and the common-mode feedback loop. Further, taking into account that the high-pass pole at $p_{1}$ is located at low frequencies, it is assumed that the bandwidth can be approximated as $BW=p_{2}/2\pi$. The input-referred rms noise $v_{rms}$ is calculated by using the expression [42]:

(3) $$v_{rms}=\frac{1}{M_{bg}}\sqrt{\gamma \cdot (BW_{Rf}\cdot S_{Rf}+\sum_{i}BW_{OTA,i}\cdot S_{OTA,i})}$$

where $BW_{Rf}$ and $S_{Rf}=4KTR_{f}$ are the equivalent noise bandwidth and the noise Power Spectral Density (PSD) of the feedback resistor $R_{f}$, respectively; and $BW_{OTA,i}$ and $S_{OTA,i}$ are the corresponding parameters for the *i*-th OTA in the LNA. In Equation (3), *γ* amounts 2 for fully-differential topologies and 1 in the case of single-ended structures. The input differential pairs of the OTAs are assumed to operate in deep weak inversion and, hence, $S_{OTA}$ is approximately given by [42]:

(4) $$S_{OTA}\approx \frac{4KT\cdot n}{2g_{m}}\left(1+\eta \right)$$

where $g_{m}=I_{b}/nU_{t}$, *n* is the transistor slope factor and *η* is a noise excess factor which depends on the OTA transistor implementation.

Based on Table 1, different conclusions can be derived regarding the performance of the different LNA topologies.

### 2.1. CFN Topology

In this simple architecture, the high-pass pole frequency is obtained by the feedback resistor ($R_{f}$) and capacitor ($C_{f}$), whereas the low-pass pole frequency is determined by the $OTA_{1}$ response. The midband gain is given by the capacitor ratio $C_{i}/C_{f}$, as long as the OTA DC gain is much higher than $M_{bg}$ (note that the feedback factor *β* can be approximated by the inverse of $M_{bg}$). Given that the required mid-band gains for neural applications are relatively high ($M_{bg}$~ 45 dB), cascode OTAs able to provide DC gains above 60 dB must be used. Under low voltage supply conditions, as it is typically found in neural recording interfaces, the use of telescopic OTAs is practically ruled out due to output swing considerations and, hence, folded-cascoded or current mirror topologies are conventionally employed at the price of considerably increasing the excess noise (η) and supply current (*k*) factors of the OTA [2,17,37]. For instance, assuming a differential (γ=2) folded-cascode OTA topology as shown in Figure 3a, a transistor slope factor *n* around 1.8, and typical factors η∼1.5, k∼4.4, a NEF above 5.5 is obtained in this topology. Current scaling [37] and current splitting [17] techniques applied to the folded-cascode OTA, together with the use of degeneration resistances at the sources of transistors $M_{3}$ and $M_{4}$, have been proposed to reduce the NEF value.

### 2.2. MIFN Topology

In this approach, the high-pass roll-off of the bandpass characteristic is implemented by an active integrator placed in a feedback path around $OTA_{1}$ [3]. The low-pass corner frequency is again determined by the frequency response of $OTA_{1}$, and the midband gain is directly given by the DC gain of this amplifier. This feature allows high midband gains without resorting to large capacitor ratios, however, strong variations in $M_{bg}$ can be expected due to technology process deviations. Given that the DC gain requirements for both OTAs are not very demanding ($A_{o1}\approx M_{bg},\;A_{o2}\gg 1$), simpler OTA topologies than in the CFN approach can be used. A good choice for $OTA_{1}$ is the cascode stage of Figure 3b which can obtain DC gains in the order of 50dB without impacting neither noise nor power consumption performance (in [3] a current mirror amplifier is employed). An even simpler structure can be used for $OTA_{2}$ as, for instance, the stage of Figure 3c.

**Figure 4:**
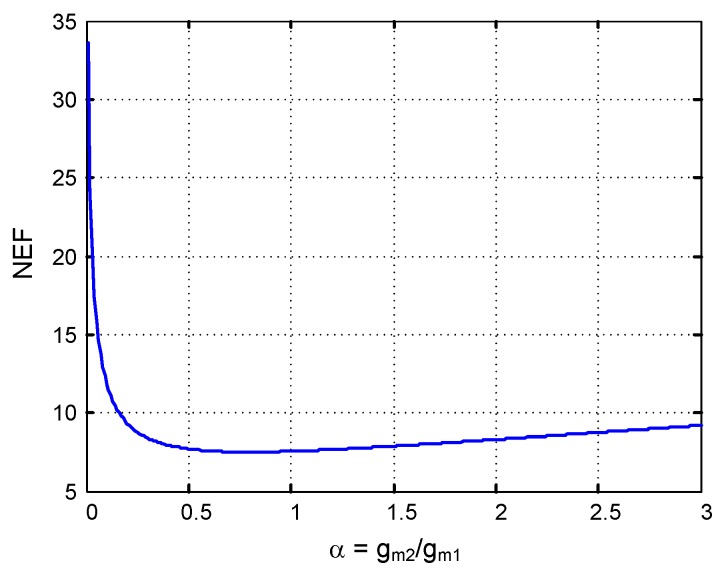
NEF *vs* transconductance ratio in the MIFN topology.

Figure 4 plots the NEF of MIFN topology in terms of the transconductance ratio *α*, assuming practical values for the OTA parameters ($\eta_{1,2}∼0.7$, $k_{1,2}∼2$). As can be seen, a minimum NEF value of about 7.5 is obtained for *α* values around unity. Hence, the MIFN topology usually presents worst noise performance than CFN, mainly because of the power consumption requirements of the second OTA. A similar conclusion can be extracted for the area requirement since large $C_{i}$ and $C_{l}$ capacitors are required to keep the input-referred noise low ($C_{i}$ amounts 35 pF in Figure 4). Further, a decoupling circuit must be used for blocking the dc offsets from the electrode-tissue interface.

### 2.3. CAFN Topology

In this architecture, the midband gain is obtained by two capacitor ratios ($C_{2}/C_{1}$ and $C_{3}/C_{4})$ using a second capacitive amplifier in the feedback loop [6]. As shown in Table 1, capacitor $C_{3}$ must be large in order to reduce the input-referred noise. In practice, this translates into a high $C_{3}/C_{4}$ ratio which forces $C_{2}/C_{1}$ to take low values for a given midband gain specification. This implies that $\beta_{1}\ll \beta_{2}$, so that factor $\chi_{2}$ can be usually neglected. Accordingly, the input-referred noise expression for the CAFN topology can be simplified to that of a CFN structure. Furthermore, since a high gain topology must be selected for $OTA_{1}$ (a folded cascode amplifier was suggested in [6] and considered herein), NEF values similar to those achievable with the CFN topology are obtained. As in the MIFN approach, $OTA_{2}$ has less impact on the noise performance of the LNA and a simpler amplifier can be used as long as it satisfies condition $A_{o2}\cdot \beta_{2}\gg 1$. The cascode stage of Figure 3b, is herein considered for $OTA_{2}$.

### 2.4. OLN Topology

An open-loop OTA is used in this approach to directly amplify the neural signal [38]. The high-pass pole frequency is determined by an input decoupling capacitor $C_{i}$ together with a resistor $R_{f}$ which in turn sets the input common-mode voltage of the OTA. The low-pass corner frequency is again determined by the OTA response. In spite of its simplicity, the midband gain is subject to large variations since it is determined by the OTA DC gain. In addition, the noise contributed by the input resistor is directly amplified to the output and it may become dominant in the total input-referred rms noise (term $A_{o}/C_{i}$ in the expression included in Table 1). Hence, the achievable NEF value depends on the midband gain and the input decoupling capacitor ($C_{i}$). Roughly speaking, the lower the NEF value targeted, the larger the input decoupling capacitors required. Regarding the OTA implementation, it is convenient to have a *β* value close to unity in order to avoid a substantial signal attenuation at the input of the amplifier. Seeking to suppress the Miller multiplication of the input pair $C_{GD}$ which would drastically increase the parasitic capacitance $C_{pi}$, the cascode amplifier of Figure 3d offers a good trade-off between input signal attenuation and output swing.

### 2.5. MCCFN Topology

This architecture is similar to the CFN topology except that the OTA is implemented by means of two amplifier stages. In some realizations a Miller capacitor $C_{c}$ (see Figure 2e) is used to guarantee stability by moving non-dominant poles of the LNA to higher frequencies [18] but, in others, no Miller compensation is employed [8,39]. The MCCFN topology offers a good trade-off between output swing, DC gain, noise and power consumption. The main reason is the degree of freedom introduced by $OTA_{2}$, which determines the output swing of the LNA with little impact on its noise performance and power consumption. This second stage also relaxes the DC gain requirement for the first stage. Indeed, in practical implementations, no cascoding techniques are used and $OTA_{1}$ is implemented by the current reuse stage in Figure 3e. This simple circuit is able to nearly double the transconductance of $OTA_{1}$ for the same tail current and, hence, a substantial reduction on the current factor $k_{1}$ can be expected; essential to lower the NEF value [9]. For $OTA_{2}$, a wide output swing structure such as Figure 3c is typically used [18]. Altogether, assuming this circuit configuration and taking practical values for the OTA parameters *η* and *k*, the minimum theoretical NEF would be around 2–3 [18].

## 3. Proposed LNA Architecture

Similar to the MCCFN approach, the proposed LNA also uses two amplifier stages, however, instead of applying a pole splitting technique to move non-dominant poles to higher frequencies, it employs feedforward compensation to create a double pole in the low-pass corner of the bandpass characteristic.

**Figure 5:**
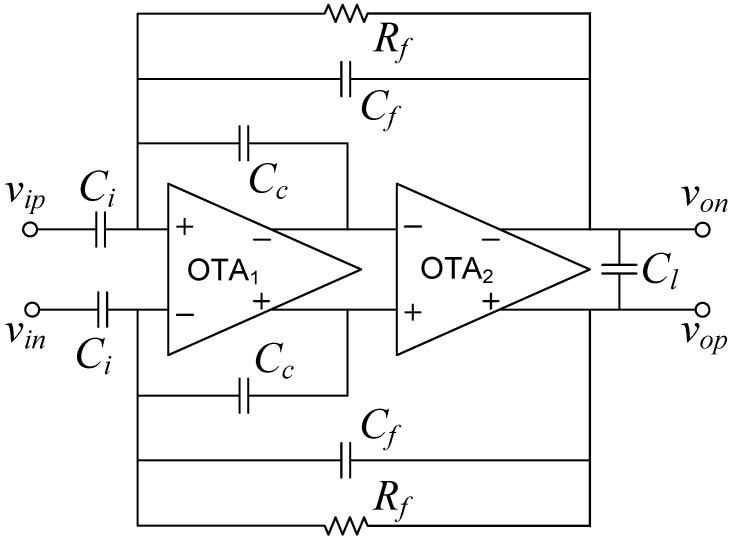
Feedforward Compensated Capacitive feedback network LNA schematic.

Figure 5 shows the schematic of the proposed Feedforward Compensated Capacitive feedback network (FCCFN) LNA, in which compensation capacitors are placed around $OTA_{1}$. Using again single-pole networks for the OTAs, it can be found after a routine small signal analysis of the LNA that its transfer function presents one zero in the origin and three poles, as well as two additional zeros at high frequencies (typically in the order of MHz). Assuming that the transconductance ratio $\alpha =g_{m2}/g_{m1}$ satisfies

(5) $$\begin{array}{c} \alpha \gg \frac{C_{f}}{A_{o1}C_{c}} \end{array}$$

and

(6) $$\begin{array}{c} \alpha \ll \frac{A_{o2}C_{t2}}{C_{t1}} \end{array}$$

the midband gain of the LNA amounts $M_{bg}\approx C_{i}/C_{f}$, the high-pass pole of the passband can be approximated as,

(7) $$p_{1}\approx \frac{-1}{R_{f}C_{f}}$$

and the two remaining poles can be made to coincide at $p_{2}^{\left(double\right)}$ to define the low-pass corner of the bandpass characteristic,

(8) $$p_{2}^{\left(double\right)}\approx \frac{-2C_{f}g_{m2}}{C_{eq}C_{f}/A_{o1}+C_{c}\left(C_{f}+C_{t2}\right)}$$

as long as the following condition holds,

(9) $$\alpha \approx \frac{C_{\alpha }^{4}}{4C_{f}C_{c}\left[\frac{C_{eq}C_{f}}{\beta_{c}}+C_{t1}\left(C_{f}+C_{t2}\right)\right]}$$

where $\beta_{c}=C_{c}/\left(C_{c}+C_{t1}\right)$, $C_{\alpha }^{2}=C_{eq}C_{f}/A_{o1}+C_{c}\left(C_{f}+C_{t2}\right)$ and the remaining parameters and capacitances take the same expressions as for the MCCFN LNA (see Table 1).

**Figure 6:**
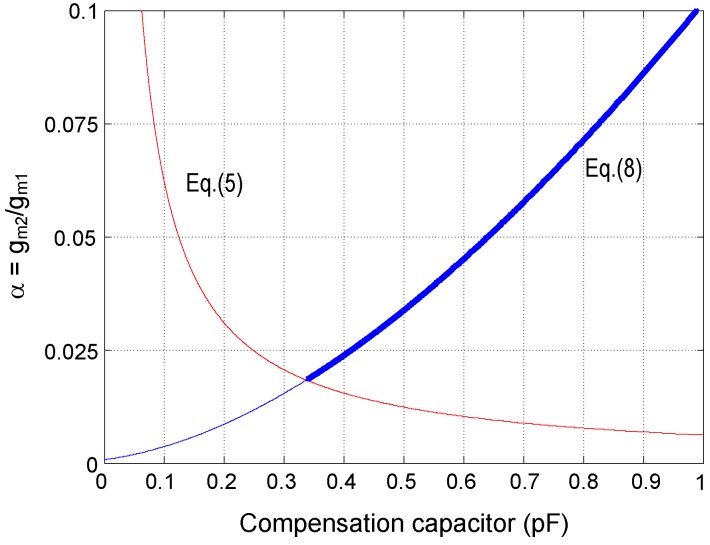
Transconductance ratio versus compensation capacitor in the FCCFN topology.

Figure 6 plots the constraint Equation (9) in terms of the compensation capacitance $C_{c}$ for a typical configuration of the LNA (parameters are shown in Table 2). In the same plot, the approximation in Equation (5) is represented assuming that *α* is 20 times larger than $C_{f}/\left(A_{o1}C_{c}\right)$—this assumption guarantees negligible errors in the pole expressions in Equations (7) and (8). The approximation in Equation (6) only imposes an upper limit on the transconductance ratio which can be hardly reached for practical $C_{c}$ values, so it is not plotted. Valid *α* values are represented with a thick trace. In order to not increase the total area occupation of the LNA, a $C_{c}$ capacitance of about 0.4 pF is a reasonable choice giving rise to α≈0.021 for this particular configuration.

In order to evaluate the noise performance of the proposed LNA, it will be assumed that the noise contribution of $OTA_{2}$, attenuated by the gain of the first stage, is negligible. Hence, taking into account that the equivalent noise bandwidth of the feedback resistor $R_{f}$ and $OTA_{1}$ can be approximated as

(10) $$BW_{Rf}\approx \frac{1}{4R_{f}C_{f}}\;\;\;\;BW_{OTA,1}\approx \frac{{\left(\beta C_{c}+C_{f}\right)}^{2}\cdot g_{m2}}{4\beta^{2}C_{f}C_{\alpha }^{2}}$$

and using Equations (3) and (4), the input-referred rms noise $v_{rms}$ is calculated as

(11) $$v_{rms}\approx \sqrt{\frac{kT\gamma }{M_{bg}C_{i}}\left(1+\frac{{\left(\beta C_{c}+C_{f}\right)}^{2}\alpha n\left(1+\eta_{1}\right)}{2\beta^{2}C_{\alpha }^{2}}\right)}$$

which can be reduced by increasing the input capacitance $C_{i}$ or the mid-band gain. Additionally, taking into account that the 3 dB-bandwidth of the LNA, assuming a double pole at $p_{2}^{\left(double\right)}$, is given by

(12) $$BW=\frac{F\cdot p_{2}^{\left(double\right)}}{2\pi }$$

where $F=\sqrt{\sqrt{2}-1}$, the noise efficiency factor NEF, defined in Equation (2), can be approximated as

(13) $$NEF\approx n\sqrt{\frac{\gamma k_{1}\left(1+\eta_{1}\right)}{4F}}$$

where it is assumed that the second term inside the parentheses in Equation (11) is much larger than unity and that $\beta C_{c}\ll C_{f}$, as occurs in practical situations. Equation (13) reveals the benefits of using a 40 dB/dec magnitude roll-off at the low pass corner of the LNA bandpass characteristic. Assuming the same OTA parameters than in the MCCFN topology, the most suitable LNA for NEF reduction reviewed in Section 2, the proposed FCCFN approach is able to further reduce the noise efficiency factor by about 15%. This point will be further corroborated in the next section.

**Figure 7:**
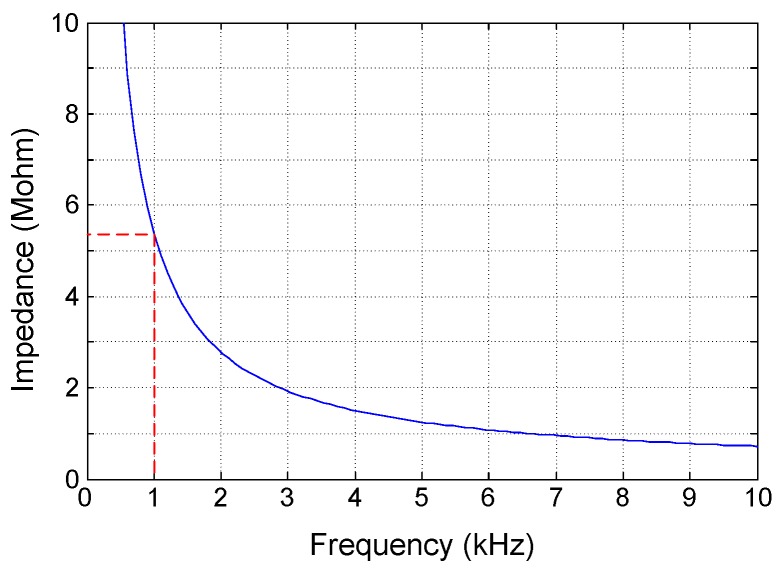
Input impedance of the FCCFN topology.

Similar as for the CFN, CAFN, OLN and MCCFN, the input impedance of the proposed FCCFN topology is dominated by the input capacitance $C_{i}$. This is illustrated in Figure 7 which represents $Z_{in}$ in terms of frequency for the parameters in Table 2. Note that at 1 kHz, the input impedance is about 5.3MΩ, well above $Z_{1kHz}$ of commercial microelectrodes [29].

**Table 2:** Parameters in a typical configuration of the FCCFN LNA.

Parameter	Values
$C_{i}\;/\;C_{f}$ (pF)	30.0 / 0.125
$R_{f}$ (GΩ)	6.4
$C_{p1i}/\;C_{t1}/\;C_{t2}/$(pF)	1.8 / 3.0 / 5.5
$A_{o1}/\;A_{o2}$	410 / 340
$f_{p1}/\;f_{p2}$ (kHz)	0.2 / 7.0

## 4. Sizing Procedure

A synthesis procedure for the transistor-level sizing of the proposed FCCFN LNA topology has been developed. The aim is to minimize the NEF factor of the structure for given specifications on the bandpass characteristics ($M_{bg}$, $p_{1}=2\pi f_{hp}$ and $p_{2}=2\pi f_{lp}$), maximum tolerable input-referred noise $v_{rms}^{max}$, and maximum active area occupation $Area^{max}$. The procedure combines a simulated-annealing optimization algorithm [43] with a set of Matlab routines for performance evaluation which make use of accurate estimations of MOS-related parameters. Analytical equations obtained in Section 3 are used for evaluation while MOS parameters are extracted from look-up tables obtained from batches of ${\mathrm{Spectre}}^{\mathrm{TM}}$ simulations in the selected technology [44]. Design variables of the synthesis procedure include the load capacitor of the LNA $C_{l}$, the feedback capacitor $C_{f}$, the inversion coefficients $IC_{1}$ and $IC_{2}$ of the input transistors of $OTA_{1}$ and $OTA_{2}$, respectively.

**Figure 8:**
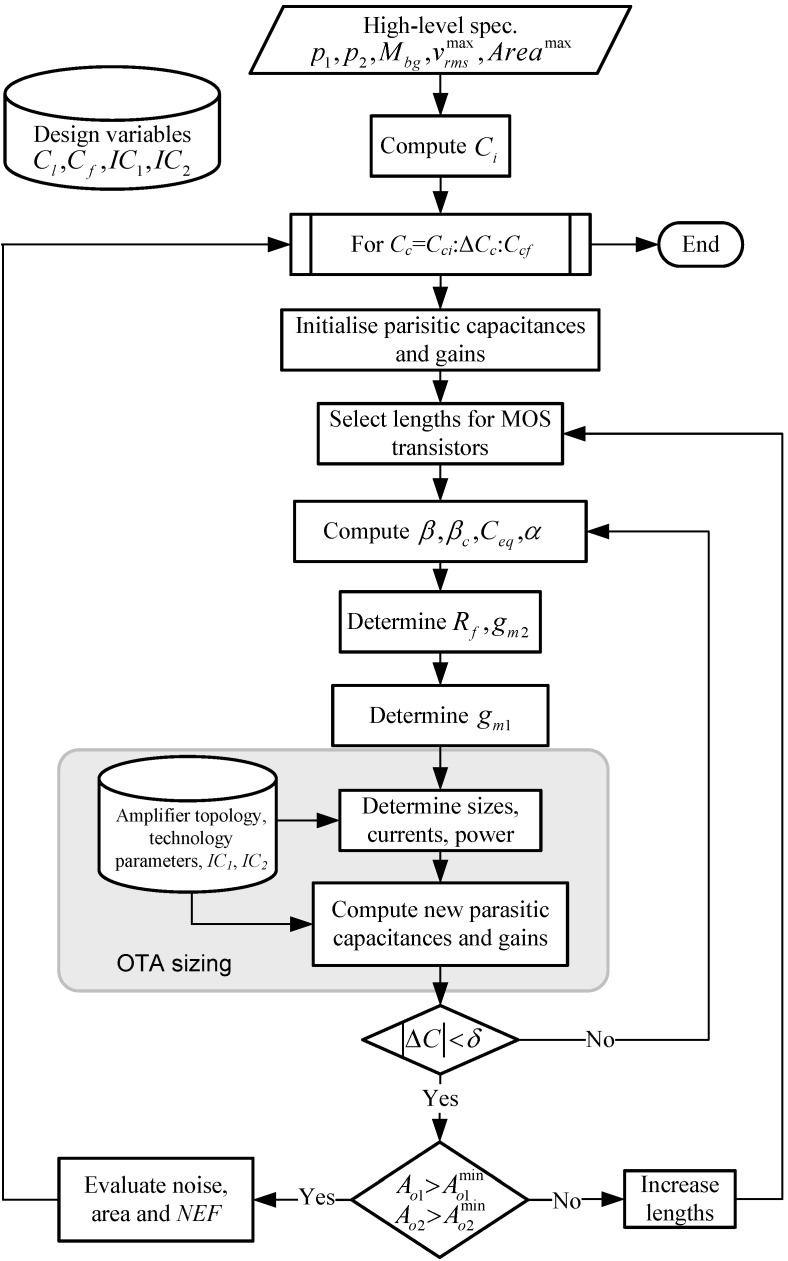
Proposed sizing procedure.

The LNA sizing procedure is illustrated in Figure 8. It starts by computing the sampling capacitor $C_{i}$ according to the required mid-band gain $M_{bg}$ and the specified feedback capacitor $C_{f}$. Then, a computational loop with the compensation capacitor $C_{c}$ as running variable is accessed. Bound values ($C_{ci}$ and $C_{cf}$) and discrete increments $\Delta C_{c}$ are user-defined. At each iteration, a new configuration (new transistor sizes and biasing currents) is obtained. If the input-referred noise ($v_{rms}<v_{rms}^{max}$) and active area constraints ($area<Area^{max}$) are satisfied, the corresponding NEF, power consumption and silicon area are stored. Otherwise, the configuration is rejected. When the loop stops, the routine selects that configuration with the lowest NEF as the final outcome of the algorithm.

Each iteration in the aforementioned loop starts by guessing initial values for the parasitic capacitances, the finite DC-gains of both OTAs and the lengths for the MOS transistors. In the case of the input transistors of the OTAs, lengths well above the minimum channel length offered by the technology are assumed in order to make the impact of flicker noise negligible as compared to the thermal noise contribution. Afterward, the feedback factors *β* and $\beta_{c}$, the equivalent input load $C_{eq}$, and the transconductance ratio *α* are calculated. Then, the values of the feedback resistor $R_{f}$ and the transconductance of the OTAs are obtained from Equation (7), Equation (8) and Equation (9), respectively. In practice, full length expressions instead of the approximated equations disclosed in the previous section are used for the sake of increased accuracy. Using this set of parameters, together with the previously planned inversion coefficients, the sizes, currents and bias voltages of the OTA MOS transistors are calculated using technology parameters (see [44] for details) and, hence, the overall power and area consumption of the LNA can be estimated. In order to validate the design, parasitic capacitances are newly calculated and compared to those previously stored. If discrepancies are higher than a user-defined tolerance value, *δ*, the iterative process is repeated again until convergence is reached. If the estimated DC-gains are lower than the required ones ($A_{o1}^{min},A_{o2}^{min}$), the lengths of MOS transistors are increased and the algorithm is repeated again. In practice, only three or four iterations are needed for convergence. It is worth observing that no ad-hoc fitting parameter are needed in the sizing procedure.

**Figure 9:**
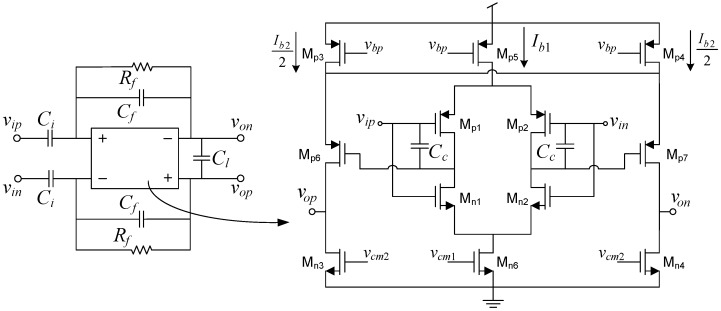
Feedforward compensated capacitive feedback network LNA transistor-level implementation.

By using this procedure, the FCCFN LNA has been synthesized at the transistor-level in a 130 nm CMOS technology for the following design specifications:.

(14) $$\begin{array}{c} \begin{array}{c} \begin{array}{ccc} M_{bg}=46\;\mathrm{dB}\;\; & f_{p1}=200\;\mathrm{Hz}\;\; & f_{p2}=7\;\mathrm{kHz} \end{array} \end{array}\\ \begin{array}{cc} area<0.05\;{\text{m}m}^{2} & \;\;\;\;\;\;\;\;\;v_{rms}<4\;\mu \mathrm{Vrms} \end{array} \end{array}$$

which are typically found in neural spike recording interfaces. Similar as in [18], $OTA_{1}$ is implemented by the current reuse stage in Figure 3e and $OTA_{2}$, uses the structure of Figure 3c. Figure 9 shows the schematic of the fully-differential LNA. The initial guess for the lengths of the input transistors of the OTAs are chosen so that the flicker noise corner frequency lies below the high pass corner $f_{p1}$. The common-mode voltage of $OTA_{1}$ is defined by a continuous-time Common-Mode Feedback (CMFB) circuit with resistive sensing which controls the tail current of $OTA_{1}$ through $M_{n6}$. An additional CMFB circuit for $OTA_{2}$, acting on transistors $M_{n3,4}$, is used to make its transconductance independent of the common-mode voltage of the first stage. The current consumption of the CMFB circuits have been accounted for in $I_{tot,1}$ through parameter $k_{1}$. Table 3 shows the sizing results together with the most relevant performance metrics obtained by electrical simulation. Observe that the structure meets the specifications in Equation (14) and obtains a NEF of about 2 with a power consumption of 1.92 *μ*W from a 1.2 V supply voltage.

**Table 3:** Sizing results for the proposed LNA.

Parameter	Value	Parameter	Value
$W_{p1,2}/L_{p1,2}$	74/3	$g_{m1}/\;g_{m2}$ (*μ*S)	35.5 / 0.76
$W_{n1,2}/L_{n1,2}$	28/8	$M_{bg}$ (dB)	46.7
$W_{p5}/L_{p5}$	13.8/4	$I_{tot,1}/I_{tot,2}$ (*μ*A)	1.5 / 0.1
$W_{n5}/L_{n5}$	4.5/4	$v_{rms}$ (*μ*Vrms)	3.62
$W_{p3,4}/L_{p3,4}$	1.3/10	NEF	2.02
$W_{n3,4}/L_{n3,4}$	0.3/10	Power (*μ*W)	1.92
$W_{p6,7}/L_{p6,7}$	0.92/4		

**Figure 10:**
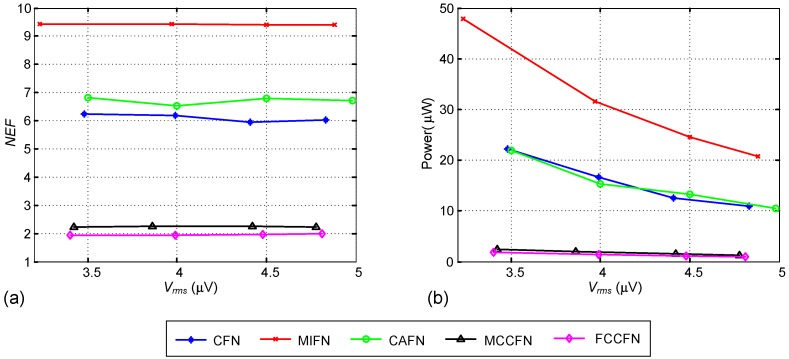
Synthesis results: (**a**) NEF; (**b**) Area; and (**c**) Power versus required $v_{rms}$.

In order to demonstrate the versatility of the sizing approach, a set of routines similar to that in Figure 8 have been developed for each of the fully-differential LNA topologies discussed in Section 2. With these routines and using the same technological process and power supply conditions, the different LNAs have been synthesized to meet the specifications in Equation (14) for different $v_{rms}^{max}$ limits. The results of the exploration are shown in Figure 10, in which the *NEF*s and power consumptions are represented against the input-referred noise. Of course, the analysis is not exhaustive, e.g., not all the OTA structures in Figure 3 have been considered for all the topologies in Figure 2. Indeed, only the transistor-level OTA configurations shown in Figure 3 have been considered. Yet, some interesting conclusions can be drawn which illustrate the triple trade-off between area, power and noise in neural recording LNAs:

1. The NEF performance of the different topologies is well aligned to the analytical results in Section 2, being the MCCFN and the proposed FCCFN topologies the best approaches.
2. The MCCFN topology uses the same OTAs than the proposed FCCFN LNA and, for the same specifications, they obtain fairly the same power consumption and active area occupation (around $0.025\;{\mathrm{mm}}^{2}$). However, as shown in Figure 10a, the NEF of the FCCFN is lower than the MCCFN case by some 15%, as anticipated in the previous section.
3. All the considered topologies are able to satisfy the performance requirements, with the exception of the OLN approach for which the obtained active area occupation is larger than specified ($0.05\;{\mathrm{mm}}^{2}$). Further, the MIFN case only satisfies the area specification for $v_{rms}^{max}$ above 4.5μV.
4. The power consumption of the different LNAs increases as the target noise level decreases. This is particularly noticeable in the CFN, MIFN and CAFN cases for which the area occupation constraint imposes higher biasing currents for given transconductance values in the OTAs.

## 5. Experimental Results

**Figure 11:**
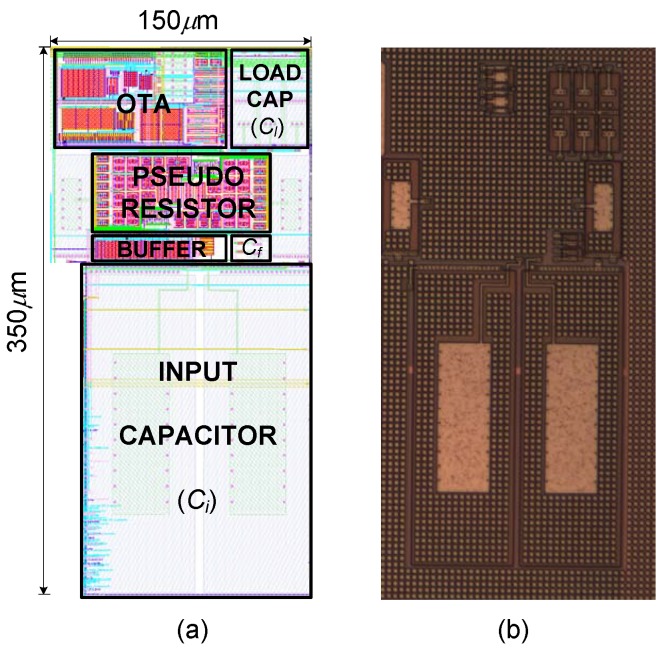
LNA implementation: (**a**) layout; (**b**) microphotograph.

A prototype of the FCCFN LNA, with the sizes detailed in Table 3, has been fabricated in a 130 nm standard 2P6M CMOS technology. Figure 11 shows the microphotograph of the LNA, together with a detailed view of the layout.

**Figure 12:**
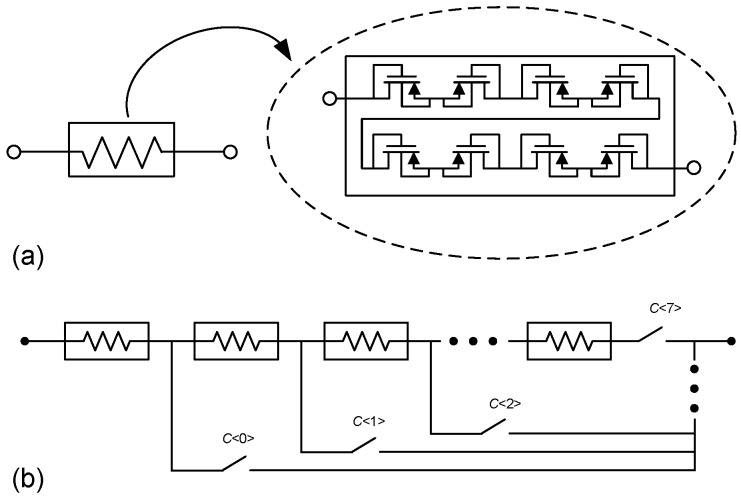
Feedback pseudoresistor implementation: (**a**) unit element; (**b**) programmable structure.

As Table 2 shows, very large feedback resistances are needed to set the high-pass pole of the bandpass characteristic. To that end, pseudo-resistors based on pMOS transistors in deep subthreshold, as shown in Figure 12a, have been employed [9]. For the sake of linearity improvement, different pMOS transistors are serially connected in order to reduce the voltage drop across their terminals. Furthermore, to cope with the large spread of the equivalent resistance under PVT variations, the feedback resistor is actually a programmable structure in which different pMOS groups, as those shown in Figure 12a, can be connected in series as determined by the 3-bit control word HPC<0:2>. This is illustrated in Figure 12b, in which the control signals c<0:7> are derived from a binary to thermometric conversion of HPC. Similarly, a 2-bit control word LPC<0:1> are used to modify the output load capacitance of the LNA and, thereby, control the position of the low-pass pole. In both programming strategies, individual elements are sized so as to uniformly cover the variation ranges estimated by PVT simulations. Indeed, measurements show a tuning range for the HP pole from 15 to 232 Hz, while the LP pole can be tuned between 5.2 kHz and 10.15 kHz. These ranges clearly cover the target bandpass characteristic for spike recording expressed in Equation (14).

**Figure 13:**
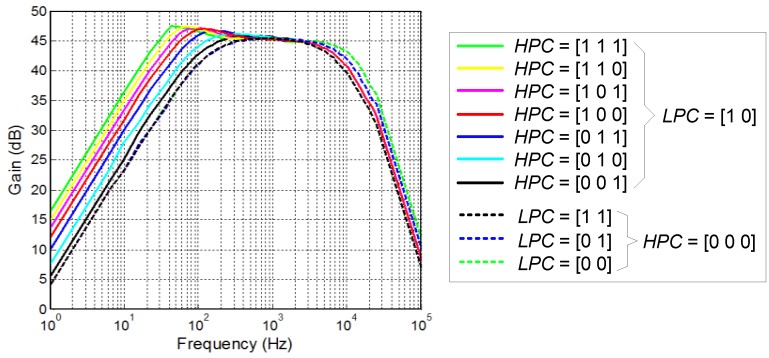
Measured LNA frequency response for different settings of the HPC and LPC tuning words.

Figure 13 shows the experimental frequency response of the LNA for all possible configurations of the tuning words HPC and LPC. After adjusting these words for neural spike recording, the high- and low-pass poles are measured to be at 192 Hz and 7.4 kHz, respectively. The mid-band gain is around 46 dB. The power supply of the LNA is nominally 1.2 V but variations of ±10% can be tolerated without significant performance deviations. In all the presented experiments, the LNA, mounted in a PCB, and the test fixtures were supplied by external batteries to avoid coupling of power line noise.

**Figure 14:**
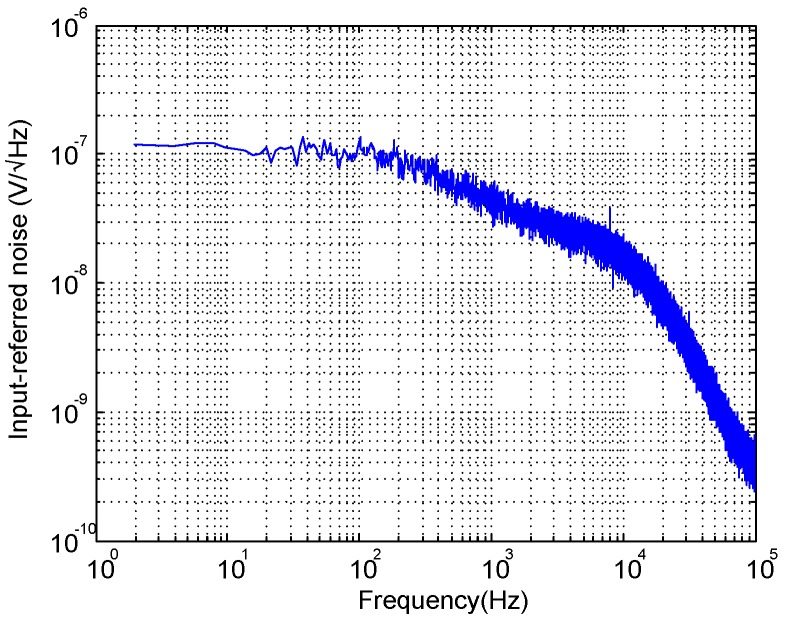
Measured LNA input-referred noise.

**Figure 15:**
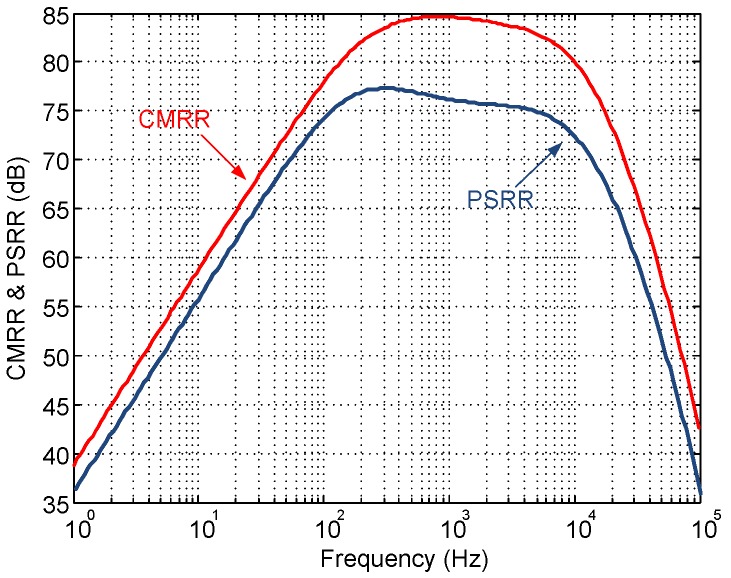
Measured LNA CMRR and PSRR.

**Figure 16:**
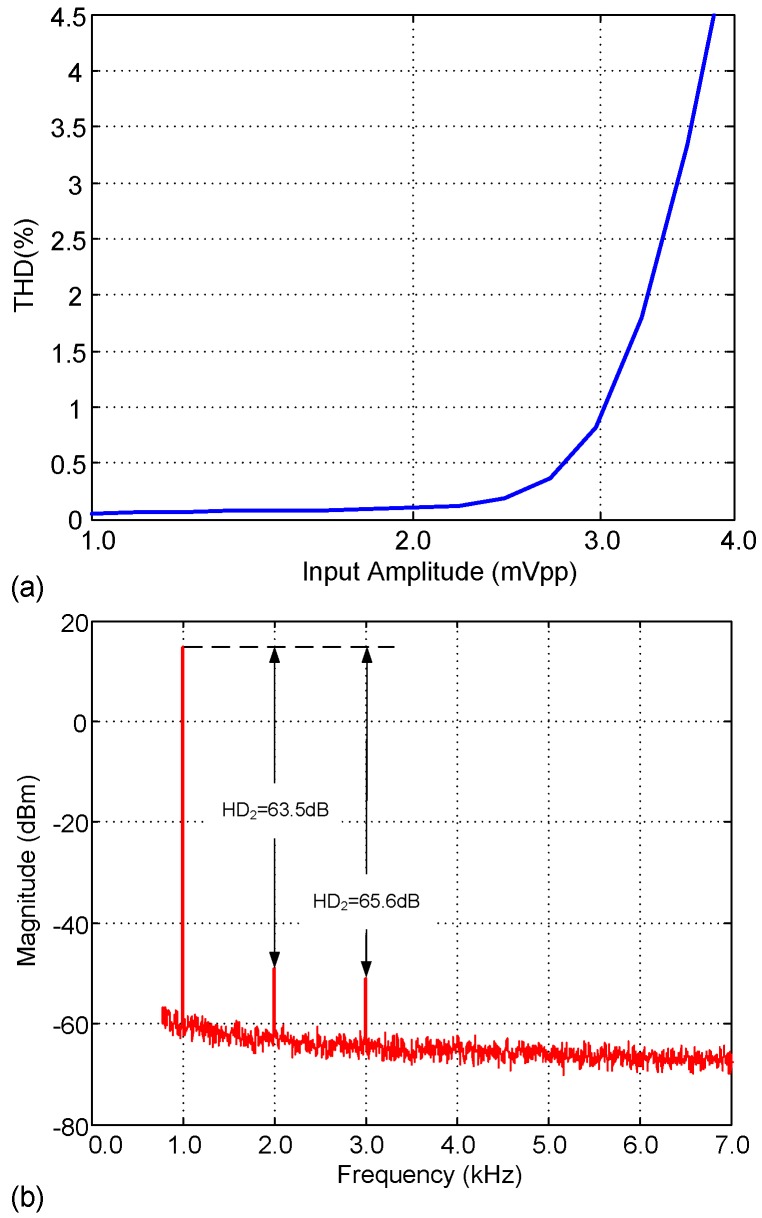
Measured LNA output distortion: (**a**) THD versus input amplitude; and (**b**) output spectrum for a 1 kHz@2 mVpp input tone.

Figure 14 shows the input-referred noise of the LNA. The measured input referred noise is $3.8\;\mu V_{rms}$, integrated from 1 Hz to 100 kHz, and $2.82\;\mu V_{rms}$ over the adjusted passband. The integrated noise and the achieved NEF are slightly larger than in Table 3 mainly because of the increased bandwidth. Figure 15 shows the experimental Common-Mode Rejection Ratio (CMRR) and Power Supply Rejection Ratio (PSRR) of the LNA, which amount 85 dB and 75 dB, respectively, in the passband. As an illustration of the linearity performance, Figure 16a plots the Total Harmonic Distortion (THD) versus the input amplitude. Note that the distortion quickly increases for input voltages above $3\;{\mathrm{mV}}_{\mathrm{pp}}$ due to the limited output swing of $OTA_{2}$. Figure 16b shows the frequency response of the LNA for a $3\;mV_{pp}$ input tone at 1 kHz. As can be seen, the second and third harmonics are more than 60 dB below the fundamental.

**Figure 17:**
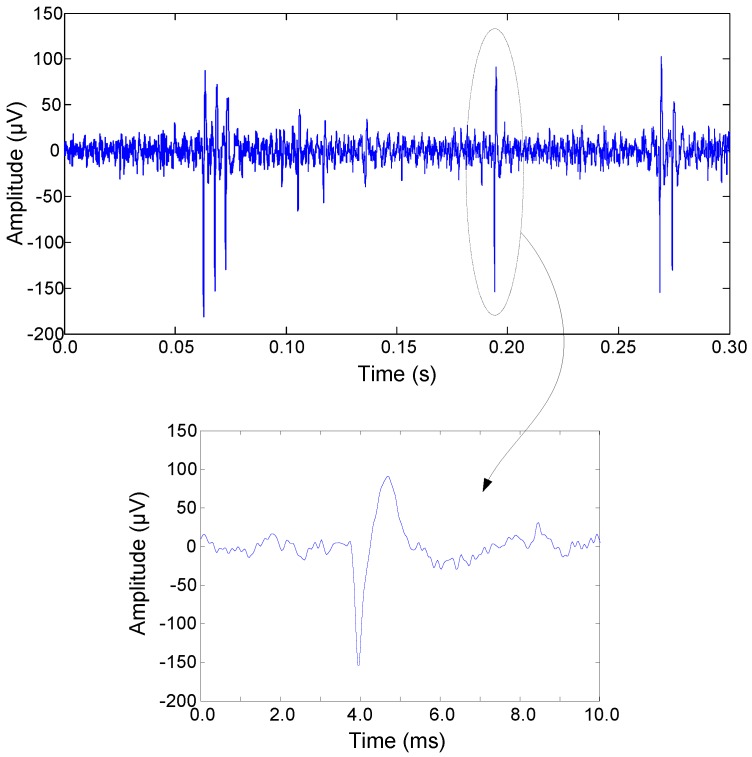
Neural spike activity recorded by the LNA by using a penetrating microelectrode. The zoom shows a single spike.

The performance of the LNA has been also validated by means of *in vivo* measurements using an animal model (adult male Long Evans rat). The experimental procedure was performed in conformance to the directive 2010/63/EU of the European Parliament and of the Council, and the RD 53/2013 Spanish regulation on the protection of animals use for scientific purposes and approved by the Miguel Hernandez University Committee for Animal use in Laboratory. A penetrating electrode (BlackRock Microsystems LLC) inserted into the visual cortex of the rat was used for probing. A large electrode placed on top of the dural surface was used for reference. The signals were transferred to the LNA by means of flat ribbon cables connected between the electrode’ connector and the PCB through row precision sockets from Samtec. Figure 17 shows a segment of neural activity recorded by the LNA as well as a zoom over one of the spikes. No significant low-frequency interference was observed during the experiment.

**Table 4:** State-of-the-Art Comparison of the LNA Measured Performance.

	[5]	[10]	[8]	[16]	[19]	[9]	[18]	[21]	This work
Voltage Supply (V)	5	1.8	1	1.8	1	1	1	1.8	**1.2**
Technology (*μ*m)	0.5	0.18	0.35	0.18	0.18	0.13	0.13	0.35	**0.13**
Fully differential	No	No	No	No	No	Yes	Yes	Yes	**Yes**
Input ref. noise ($\mu V_{rms}$)	2.2	5.6	4.43	3.5	4	1.95	2.2	2	**3.8**
Noise int. bandwidth (Hz)	N/A	1–100 k	1–12 k	10–100 k	1–8 k	0.1–25.6 k	0.1–105 k	0.1–100 k	**1–100 k**
Bandwidth (Hz)	0.025–7.2 k	98.4–9.1 k	217–7.8 k	10–7.2 k	0.38–5.1 k	23 m–11.5 k	50 m–10.5 k	0.1–6 k	**192–7.4 k**
Gain (dB)	39.5	49.52	45.7	39.4	60.9	38.3	40	52-75	**46**
CMRR (dB)	83	50	58	70.1	60	63	80	90	**85**
PSRR (dB)	85	50	40	63.8	70	63	80	78	**75**
THD	1%	1%	0.53%	1%	1%	1%	1%	N/A	**0.08%**
Input range ($mV_{pp}$)	12.4	2.4	full range	5.7	0.9	0.16	1	N/A	**3.0**
Power cons. (*μ*W)	80	8.4	1.26	7.92	0.81	12.5	12.1	8.1	**1.92**
NEF	4	4.9	2.16	3.35	1.9	2.48	2.9	1.84	**2.16**
$NEF^{2}\cdot V_{dd}$	80	43.22	4.67	20.20	3.6	6.15	8.41	6.14	**5.59**

Table 4 summarizes the performance of the LNA and compares it with state-of-the-art publications on neural recording sensors. In all cases, the reported sensors were verified *in vivo* with penetrating intracortical electrodes: [18,19] used neural probes by NeuroNexus®, [10,21] used custom assemblies and all the rest, including our proposal, were tested with Utah array. In some cases, the LNA is not specifically tailored for neural spike recording but extends the high-pass corner to lower frequencies (e.g., [5] or [9]). The commonly used Noise Efficiency Factor (NEF), defined in Equation (2), is shown as a dimension-less Figure of Merit (FoM) for comparison. Additionally, a newer FoM that reflects the employed voltage supply, $NEF^{2}\cdot V_{dd}$, is also calculated [40]. These numbers show that, compared to the rest of the presented works, the proposed design presents one of the lowest FoMs, only beated by [8,19], which are favoured in terms of power consumption by their single-ended designs, at the cost of a worst rejection to supply and common-mode variations.

## 6. Conclusions

A new LNA architecture has been presented, where the use of a two-stage OTA with a feed-forward compensation provides several advantages with respect to other approaches. The proposed architecture has been analyzed and their performance has been characterized and compared to prior art. Also, a design methodology to synthesize at transistor-level the LNA has been described. The LNA has been implemented in a 130 nm technology and experimentally validated, including *in vivo* measurements. The proposed architecture satisfactorily solves the triple trade-off between area, power and noise and, additionally, obtains excellent CMRR, PSRR and linearity performance.
